# Caligula: a neuropsychiatric explanation of his madness

**DOI:** 10.1590/0004-282X-ANP-2020-0358

**Published:** 2021-05-08

**Authors:** Jesús David CHARRY-SÁNCHEZ, Alberto VELEZ-VAN-MEERBEKE, Leonardo PALACIOS-SÁNCHEZ

**Affiliations:** 1 Universidad del Rosario, Escuela de Medicina y Ciencias de la Salud, Grupo de investigación en Neurociencia NeURoS, Centro Neurovitae, Bogotá, Colombia. Universidad del Rosario Universidad del Rosario Escuela de Medicina y Ciencias de la Salud Grupo de investigación en Neurociencia NeURoS Bogotá Colombia

**Keywords:** Lead Poisoning, Psychotic Disorders, Status Epilepticus, Intoxicación por Plomo, Trastornos Psicóticos, Estado Epiléptico

## Abstract

Gaius Julius Caesar Augustus Germanicus, better known as Caligula, (12 CE to 41 CE) was the third Roman emperor and ruled only four years. Throughout his life he experienced several traumatic events, and, in addition, historians mention some premorbid conditions that could cause him to become the monster that most historians know today. When Caligula was 25 years old, he suffered a near-fatal illness that turned his story around. One possible cause was lead poisoning due to the high consumption of wine, which contained lead, by Roman patricians. On the other hand, it is plausible that Caligula experienced epilepsy that began in childhood, later experienced status epilepticus in 37 CE, which triggered an epileptic psychosis with the consequent psychopathic and paranoid changes that led him to the madness noted by historians.

## RESUMEN

Gaius Julius Caesar Augustus Germanicus, mejor conocido como Calígula**,** (12 C.E. - 41 C.E.), fue el tercer emperador romano y gobernó durante apenas cuatro años. A lo largo de su vida, experimentó diferentes eventos traumáticos y, adicionalmente, algunos historiadores mencionan condiciones premórbidas que pudieron causar que se convirtiera en el monstruo que los historiadores conocen hoy. Cuando Calígula tenía 25 años, sufrió una enfermedad casi fatal que le dio un giro considerable a su historia. Una posible causa es intoxicación por plomo, debido al excesivo consumo de vino, el cual contenía considerables niveles de este metal. Por otro lado, es posible que Calígula cursara con crisis epilépticas de inicio en la infancia. Posteriormente, en el año 37 C.E. experimentara un estado epiléptico, el cual desencadenaría una psicosis epiléptica, dejando secuelas que iban a desencadenar la locura de la que muchos historiadores hablan.

## A BRIEF HISTORICAL OVERVIEW

Caligula was born in Anzio on August 31, 12 CE, and was the son of Agrippina and Germanicus. From an early age, he experienced several traumatic events that may have influenced his development in childhood and adulthood^1^^,^^2^^,^^3^. During his childhood, at the age of 18 months, he was left with his great-grandfather, Augustus, owing to his health conditions. Later, when he was 2, Caligula began to travel with his parents to military campaigns^3^. According to Suetonius, on one of his parents’ military campaigns, Caligula suffered from the falling sickness, the name for epilepsy^1^^,^^3^. At the age of 3, his mother made him a small uniform, due to the size of his boots, his father’s soldiers called him Caligula, the diminutive of caliga^2^^,^^4^. When Caligula was 7 years old, his father was assassinated on a military excursion, and, after this incident, Caligula returned with his family and lived with his grandmother, Livia. Seven years later, Tiberius executed Agrippina and two of Caligula’s brothers, and exiled his sisters. Despite this, historians have noted that Caligula showed no feelings when his relatives died^1^^,^^2^^,^^3^.

At the age of 20, Tiberius brought him to Capri to live with him and his nephew Gemellus and declared them co-heirs^1^^,^^2^. By March of 37 CE, Tiberius died, and Caligula was chosen as emperor of Rome. During the first months, Romans loved Caligula because of his exceptional work on the government^1^^,^^2^^,^^3^. In October of the same year, when he was 25 years, Caligula experienced a severe illness. Some historians argued that Caligula had a history of seizures and mental illness, some other sources have also suggested that he suffered from a “disorder of intellect” and experienced anxiety and insomnia. However, regardless of the nature of the disease, most historians have concluded that Caligula’s illness affected his mental health and caused the personality changes that he experienced after his recovery^1^^,^^2^^,^^3^^,^^5^^,^^6^^,^^7^^,^^8^^,^^9^^,^^10^. After his near-fatal illness, he became an alcoholic, believed he was a deity, and, moreover, he became paranoid about the risk of assassination, which led him to sentence Gemellus and Macro to death^1^^,^^2^^,^^3^^,^^5^^,^^6^^,^^7^^,^^8^^,^^9^^,^^10^^,^^11^. Finally, after all the eccentric behavior Caligula displayed in his short period of government, he was assassinated in 41 CE at the age of 28 years^1^^,^^2^.

## FROM WHICH NEUROLOGICAL AND PSYCHIATRIC ILLNESSES DID CALIGULA SUFFER?

Historians have proposed several possible conditions and illnesses that Caligula may have had during his life. These conditions are summarized in Table 1.

**Table 1.** Possible illnesses of Caligula.

Illnesses	References
Epilepsy	Baratta & Halleguen, 2009^6^; Sidwell, 2010^3^; Benediktson, 1989^7^; Camargo et al., 2018^8^; Demetrioff, 2018^11^; Sandison, 1958^2^
Insomnia	Sidwell, 2010^3^; Camargo et al., 2018^8^; Demetrioff, 2018^11^; Katz, 1972^9^; Sandison, 1958^2^
Encephalitis	Sidwell, 2010^3^; Camargo et al., 2018^8^; Demetrioff, 2018^11^; Sandison, 1958^2^
Lead poisoning	Gilfillan, 1965^14^; M R Reese, 2019^1^; Mackie et al., 1975^10^
Neurosyphilis	Baratta & Halleguen, 2009^6^
Bipolar disorder	Baratta & Halleguen, 2009^6^; Sidwell, 2010^3^
Hyperthyroidism	Baratta & Halleguen, 2009^6^; Sidwell, 2010^3^; Benediktson, 1989^7^; Katz, 1972^9^
Anxiety disorder	Baratta & Halleguen, 2009^6^; Sidwell, 2010^3^; Benediktson, 1989^7^; Katz, 1972^9^; Sandison, 1958^2^
Personality disorder (sociopathy)	Baratta & Halleguen, 2009^6^; Sidwell, 2010^3^; Benediktson, 1989^7^
Schizophrenia	Baratta & Halleguen, 2009^6^; Sidwell, 2010^3^; Benediktson, 1989^7^
Alcoholism	Sidwell, 2010^3^; Benediktson, 1989^7^; Demetrioff, 2018^11^; Sandison, 1958^2^

One of the most common suggestions for Caligula’s illness is encephalitis. In modern times, even with adequate treatment, encephalitis has a high mortality rate that varies between 8.8 and 12.8% depending on its etiology^12^^,^^13^. Additionally, the disease can lead to serious neurological sequelae^13^. For that reason, the probability that Caligula had encephalitis is low. Because of the previously mentioned, this review will focus on lead poisoning and epilepsy.

## LEAD POISONING

Lead poisoning is known as “saturnism” or “plumbism”, and its clinical manifestations include cognitive disorders, fatigue, irritability, anxiety, vomiting, and anorexia. Its name is attributed to the god Saturn who shared some of these characteristics, particularly a melancholic temperament and sullen behavior^12^.

In ancient Greece and Rome, a syrup named “sapa” was added to wine during its preparation to improve its taste. Sapa was prepared in lead vessels in which acidified wine was boiled, which promotes the synthesis of lead acetate^13^. Several historians, including Pliny the Elder, Cato the Elder, and Columella, mention this “lead sugar” in their writings^12^.

Gilfillam mentions that wine was ingested almost exclusively by aristocrats and the emperor. Furthermore, he attributes the problematic behavior of some Roman emperors to lead poisoning. This can be supported by Mackie et al., who analyzed human bones from ancient Rome and found that the bones of aristocrats contained higher levels of lead than those of slaves^10^. Many people have therefore considered this as one of the factors that led to the fall of the Roman Empire^14^. However, Gilfillan's work has been criticized by many disciplines, who point out that some other factors contributed to the fall of Rome^13^.

## EPILEPSY

Most historians agree that epilepsy is the most likely candidate for Caligula’s illness. There are several details in Caligula’s biography that support this argument. It has been suggested that members of the Julius family suffered from epilepsy^2^^,^^8^. Additionally, several historians point out that, during his childhood, Caligula had episodes of sudden falls in which he lost consciousness and had difficulty remaining upright^3^^,^^7^^,^^11^. Analyzed from a modern perspective, these episodes may indicate atonic seizures.

Furthermore, Benediktson citing Suetonius reports that Caligula sometimes became febrile, which is a symptom concomitant with the onset of temporal epilepsy^7^. However, Demetrioff points out that these episodes were not frequent during Caligula’s adolescence. This may not necessarily be evidence against the epilepsy theory, because people with epilepsy can lead a normal life during interictal periods^11^.

Regarding Caligula’s background, it is possible that he experienced status epilepticus in 37 CE, which left him with emotional, behavioral, and cognitive sequelae. After this episode, he showed constant mood swings with irascibility or unmotivated laughter, lack of impulse control, perverse behaviors, hypersexuality, and sadism, and was terrified by thunder and loud noises^2^^,^^6^. Caligula also suffered from severe insomnia and could not sleep more than three hours per night. Furthermore, he experienced delusions of grandeur, paranoid episodes, and strange behaviors, such as when he ordered his troops to collect seashells from the shore^2^.

All these symptoms fit what in modern epileptology is known as epileptic psychosis. This condition has a prevalence of 5.6 to 5.9% and can occur with any type of epilepsy. However, its prevalence increases to 9.3% in people with temporal lobe epilepsy^15^^,^^16^. One of the first onset symptoms is insomnia, something frequently mentioned in accounts of Caligula^2^^,^^3^^,^^9^^,^^11^^,^^17^. Epileptic psychosis can also be characterized by symptoms of depression, delusions, manic psychosis, strange thoughts, and behavior^17^^,^^18^.

It is plausible that Caligula experienced epilepsy that began in childhood, possibly with febrile seizures and later dialeptic or cognitive seizures. Caligula later experienced status epilepticus in 37 CE, which triggered an epileptic psychosis with the consequent psychopathic and paranoid changes. This clinical condition may have been affected by excessive alcohol intake and lead poisoning.
